# Analysis of Transcripts Expressed in One-Day-Old Larvae and Fifth Instar Silk Glands of Tasar Silkworm, *Antheraea mylitta*

**DOI:** 10.1155/2010/246738

**Published:** 2010-05-04

**Authors:** Samita Maity, Sagar I. Goel, Sobhan Roy, Suvankar Ghorai, Swati Bhattacharyya, Aravind Venugopalan, Ananta K. Ghosh

**Affiliations:** ^1^Department of Biotechnology, Indian Institute of Technology, Kharagpur 721302, India; ^2^Department of Electrical Engineering, Indian Institute of Technology, Kharagpur 721302, India

## Abstract

*Antheraea mylitta* is one of the wild nonmulberry silkworms, which produces tasar silk. An EST project has been undertaken to understand the gene expression profile of *A. mylitta* silk gland. Two cDNA libraries, one from the whole bodies of one-day-old larvae and the other from the silkglands of fifth instar larvae, were constructed and sequenced. A total of 2476 good-quality ESTs (1239 clones) were obtained and grouped into 648 clusters containing 390 contigs and 258 singletons to represent 467 potential unigenes. Forty-five sequences contained putative coding region, and represented potentially novel genes. Among the 648 clusters, 241 were categorized according to Gene Ontology hierarchy and showed presence of several silk and immune-related genes. The *A. mylitta* ESTs have been organized into a freely available online database “AmyBASE”. These data provide an initial insight into the *A. mylitta* transcriptome and help to understand the molecular mechanism of silk protein production in a Lepidopteran species.

## 1. Introduction

The knowledge of lepidopteran biology and genetics is most advanced in the mulberry silkworm *Bombyx mori* due to the availability of extensive genome and EST sequence data [1, 2]. A recent EST sequencing project of *Spodoptera frugiperda* also describes about 35% of the potential total gene number in the organism [3]. Among the lepidopterans, the silkmoths, especially *B*.* mori*, *Antheraea pernyi*, *A*. *mylitta, *and *Antheraea assama *play an important role in rural economy of many populous developing nations. Six million people in India alone are involved in sericulture and one of the most economically important species for sericulture in India is the wild type non-mulberry silkworm, *A. mylitta*, which produces the exotic variety of silk called tasar silk [4]. This Saturniidae silkworm has four major stages in its life cycle-larva, cocoon, moth and egg. It is a tetramolter and passes through five instars. This species is endemic and distributed in different geographical regions of India in the form of different ecological races [5]. It shows variation in phenotypic traits such as fecundity, voltinism, cocoon weight, and also in its host plant preference [6]. The population of *A*. *mylitta* is declining due to reasons like deforestation, pathogenic infection and socio-economic problems. The cytoplasmic polyhedrosis virus infection alone leads to 20–30% death of larvae every year. Although tasar silk is different from other varieties of non-mulberry silk in respect to colour, lustre and strength but this silk producing insect has not been studied at molecular level to understand its various stages of development, silk production and immunity against infection. Recently, construction of an EST database for wild silkmoths consisting of *A*.* assama*, *Samia cynthia ricini* and *A*.* mylitta* has been reported [7]. But despite its economical and ecological importance, the current genomic resources for *A*.* mylitta *are limited to 1412 EST sequences from a cDNA library constructed from the fat bodies of bacteria challenged *A*.* mylitta* larvae [8]. Several antimicrobial proteins including insect lysozyme and protease inhibitor have been identified from *A*. *mylitta* [9–12]. Thus an EST sequencing project is executed by generating two cDNA libraries from *A. mylitta* and sequencing clones from these libraries to identify the genes involve in silk production as well as metabolism, and immunity. Moreover, establishment of genomic resources from different lepidopteran species will provide comparative insights that are crucial for understanding diversity and variability at multiple levels of biological organization.

We report here a diverse set of ESTs from cDNA libraries constructed from two developmentally spaced larval stages—one day old larvae and silkglands of fifth instar larvae. Two thousand four hundred and seventy six good quality ESTs have been obtained and assembled into 648 clusters, annotated and classified according to Gene Ontology hierarchy. Finally a freely available online database “AmyBASE” has been created for easy access to all these ESTs.

## 2. Materials and Methods

### 2.1. Materials

One-day-old and fifth instar *A*.* mylitta* larvae were collected from the silk farms of West Bengal and Jharkhand states of India.

### 2.2. cDNA Library Construction

Whole body of one day old larvae and isolated silkglands of fifth instar larvae were ground in liquid nitrogen and total RNA was extracted by guanidium isothiocyanate method [13]. mRNA was purified from the total RNA by oligo (dT)-cellulose chromatography [14] using poly (A) Quick mRNA extraction kit (Stratagene). First strand cDNA was synthesized from 5 *μ*g of purified mRNA according to the procedure of Gubler and Hoffman [15] using the cDNA synthesis kit (Stratagene or Pharmacia). Second strand synthesis was carried out using the RNaseH procedure as per the manufacturer's protocol. Subsequently, cDNAs were cloned into lambda ZapII vector and packaged into bacteriophages using the Gigapack packaging system (Stratagene). Unamplified packaged phage was titred in *E*.* coli* and then phagemids were rescued by infecting *E*.* coli* with helper phage and grown in ampicillin containing media plates as bacterial colonies.

### 2.3. EST Sequencing and Submission

One thousand six hundred bacterial colonies from the unamplified one day old larval cDNA library and one thousand seven hundred from unamplified fifth instar larval silkgland cDNA library were grown in 5 mL Luria broth (LB) at 37°C for overnight. Plasmid DNA was isolated using miniprep kit (Qiagen) and analyzed by agarose gel electrophoresis after digestion with *Eco*RI. Plasmids with inserts from 100 bp to 4 kbp (698 from one day old larval library and 722 from 5th instar larval silkgland library) were selected and sequenced from both ends using M13 forward and reverse primers and Big dye terminator (ABI), in an automated DNA sequencer (ABI 3100). The vector sequences and the ambiguous bases at the ends were trimmed using Sequencher 4.1.4 software. Sequences shorter than 120 bp were not included in the analysis which finally resulted in 2476 ESTs (1221 from one day old larval library and 1255 from silkgland library). These sequences were submitted to GenBank dbEST under the accession numbers [EG591733-EG594207, EG629961].

### 2.4. EST Assembly and Annotation

To identify ESTs belonging to the same open reading frames (ORFs), 2476 sequences were assembled using Dirty data algorithm (Sequencher 4.1.4) with 90% minimum match and 60 bp minimum overlap length. A cluster containing ≥2 ESTs was termed a contig and that containing only one sequence, a singleton. A local version of AutoFACT [16] was used to compare the clusters against a total of six databases. The European Ribosomal Database (http://www.psb.ugent.be/rRNA/index.html), [17] was searched using BLASTn; Uniprot's Uniref90 (ftp://ftp.uniprot.org/pub/databases/uniprot/uniref/uniref90/), [18] NCBI's non-redundant database (ftp://ftp.ncbi.nih.gov/blast/db/) and Clusters of Orthologous Groups (COG) (ftp://ftp.ncbi.nih.gov/Pub/COG/COG/), [19] were searched using BLASTx; and Proteins Families Database (Pfam) (ftp://ftp.ncbi.nih.gov/pub/mmdb/cdd/little_endian/), [20] and Simple Modular Architecture Research Tool (Smart) (ftp://ftp.ncbi.nih.gov/pub/mmdb/cdd/little_endian/), [21] were searched using RPS-BLAST. In each instance, a bit score cutoff of 40 was used and the top 10 BLAST hits were filtered for uninformative terms.

### 2.5. Functional Categorization of Clusters

In order to functionally categorize the clusters, the Uniprot IDs of the annotated sequences were searched against QuickGO (http://www.ebi.ac.uk/ego/), the web based browser of the Gene Ontology data bases at the EBI (Gene Ontology Consortium 2000). GO terms were assigned to the clusters in the three main GO categories: molecular function, biological process and cellular component. Each cluster was further assigned to one or more subcategories based on all the GO terms assigned to it. The complete GO mapping and subcategory assignment data can be accessed online at http://www.btc.iitkgp.ernet.in/.

### 2.6. Database Design

The database was created using the open source software MySQL to store and navigate through EST sequences and their annotation. PHP scripts connected the web-based front end to the database.

## 3. Results and Discussion

### 3.1. Generation and Assembly of *A. mylitta* ESTs

**Figure 1:**
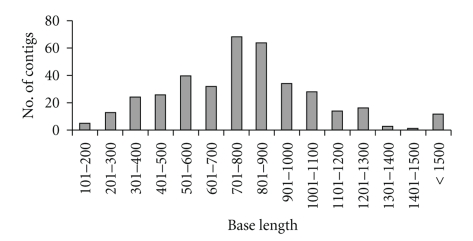
Distribution of sequence length (bp) of assembled contigs. The average length of the contigs was 774 bp.

Two independent cDNA libraries were used for sequencing. One was derived from mRNA extracted from one day old larvae and the other from the silk glands of fifth instar larvae. Insert containing 1420 clones were sequenced from both the 3′ and 5′ ends resulting in 2840 sequences. Sequencing was done from both the ends not only to eliminate sequencing errors due to a single-pass read but also to increase the probability of finding the entire coding region of a gene. The initial 2840 sequences were trimmed of vector, adaptor and low quality sequence and filtered for a minimum length of 120 bp, resulting in 2476 ESTs which were considered for further analysis. The average sequence read length was about 650 nucleotides. In order to identify ESTs belonging to the same open reading frame (ORF), sequences were assembled using Sequencher 4.1.4 assembly program. Assembly of 2476 sequences resulted in 390 contigs and 258 singletons, together making 648 clusters. Since both 3′ and 5′ sequences were treated as independent data, sequences from the same clone may be a part of two different clusters. This may be possible when clusters represent different regions of the same gene or when the same gene has similarity with two different genes. Based on the source of each cluster a set of 467 potential unigenes were identified. Two clusters were considered to be part of the same unigene when each contained sequences from one of the two ends of the same clone. The number of ESTs in the contigs varied from 2 to 127. Most of the contigs consisted of two ESTs which were primarily the sequences obtained from two ends of the same clone. The length of the contigs varied from 125 to 2723 nucleotides with an average contig length of 774 nucleotides (Figure 1).

### 3.2. Comparison to Existing *A. mylitta* ESTs in NCBI

A total of 1412 ESTs were available in the NCBI dbEST prior to the submission of our sequences [7, 8]. These ESTs were generated from the fat body of bacteria challenged larvae. In order to examine the degree of overlap between the available ESTs and our data, we queried our 648 clusters against the *A. mylitta* ESTs in NCBI dbEST using tBLASTn algorithm. About 7% (45 out of 648) of the clusters had an identity greater than 95% and can be considered to be putatively identical. Thus, about 93% (603 out of 648) of our clusters add to the existing resource of ESTs for *A. mylitta*.

### 3.3. Annotation of *A. mylitta* ESTs

**Figure 2:**
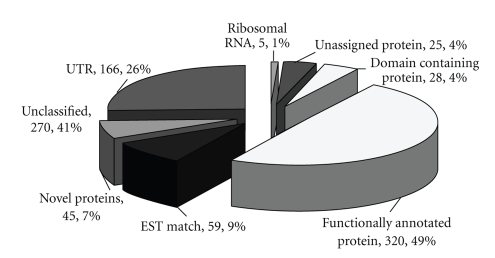
A pie-chart showing the distribution of *A. mylitta* assembled sequences by annotation classes. Section sizes are not proportional.

In order to identify putative homologues of known proteins, we annotated the clusters using AutoFACT, a fully automated and customizable annotation tool that assigns biologically informative functions to a sequence [16]. It adopts the uninformative rule [22], by which the highest scoring BLAST hit with a biologically informative description is considered informative. The most informative functional description is determined by combining multiple BLAST reports from several user-selected databases. The databases selected were European Ribosomal Database [17], Uniprot's Uniref90 [18], Clusters of Orthologous Groups (COG) [19], Proteins Families Database (Pfam) [20], Simple Modular Architecture Research Tool (SMART) [21] and NCBI's non-redundant database (http://www.ncbi.nlm.nih.gov/). Each cluster was first queried by BLAST search against the rRNA databases, followed by BLAST against protein databases (UniRef90, nr and COG) in the event of an insignificant hit to rRNA database and finally by RPS-BLAST against domain databases (Pfam and SMART) if no significant hit is obtained from the previous two BLAST searches. Thereafter each cluster was automatically assigned by AutoFACT to one of five annotation classes—“ribosomal RNA”, “functionally annotated protein”, “unassigned protein”, “domain containing protein” or “unclassified”. A sequence is classified as unassigned protein when it does not contain any known protein domain and no common informative terms are found between any of the databases, or when only uninformative hits are found. A sequence is classified as “unclassified” when no hits are found to any of the specified databases [16]. Figure 2 shows the distribution of *A. mylitta* clusters by annotation class. A total of 378 clusters were assigned annotation other than “unclassified”. A total of 270 clusters were assigned to the unclassified annotation class. This suggests that about 42% of the clusters do not have any significant homology to known rRNA or protein or domain sequences in public databases. We performed tBLASTX searches for these 270 sequences against the NCBI EST database (http://www.ncbi.nlm.nih.gov/dbEST/) using an *E*-value cutoff of 1*E* ^−06^ to assign potentially homologous sequences. Fifty-nine of these showed a significant hit with known ESTs (NCBI and other databases). We further determined that 45 of the remaining 211 sequences contained a protein coding region using the web based ESTscan tool (http://www.ch.embnet.org/software/ESTScan.html) and represented potentially novel genes. The remaining sequences that did not contain any coding regions mostly represented 3′ untranslated regions (3′ UTR). This may be due to the construction of cDNA libraries using oligo (dT) primer.

### 3.4. One-Day-Old Larvae and Silk Gland Transcript Distribution

**Figure 3:**
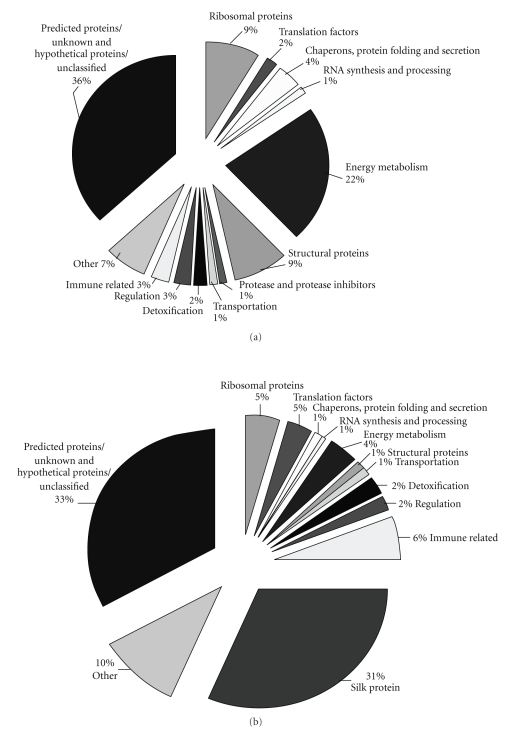
Analysis of transcriptome of one day old larvae (a) and silk gland (b).

Both libraries were unnormalized, and clones were randomly sequenced, the number of ESTs in a particular cluster could give an idea about the expression level of the particular gene. The largest contigs representing the most abundant ESTs are shown in Table 1 with their respective annotation and library information. These analyses were done depending upon BLASTX score of ESTs against NCBI nucleotide non-redundant database. The transcripts in one day old larvae and silk gland showed significant different expression pattern of genes as it was expected (Figure 3). In one day old larvae 33% transcripts were encoded for proteins involve in house keeping functions, 16% for protein synthesis and 14% for other categories. But in silk gland 52% transcripts were encoded for proteins grouped into others categories including the silk proteins, 9% transcripts for protein synthesis and 4% transcripts were encoded for house keeping functions. Both the libraries showed significant fraction of transcripts (37% of one day old larvae and 33% of silk gland) belongs to hypothetical or unclassified proteins and may lead to the identification of novel proteins.

#### 3.4.1. Genes Involve In-House Keeping Function

The proteins involved in house keeping functions included 41 energy metabolism enzymes such as cytochrome c oxidase subunits, ubiquinone dehygrogenase, ATP synthase subunit, phosphoribosyl pyrophosphate synthetase, and so forth, 24 structural proteins such as troponin, cuticular protein and tubulin, and so forth, 8 processing and degradation proteins such as proteases and ubiquitin, and so forth, 7 transportation proteins such as vacuolar type H^+^ translocating inorganic pyrophosphate, amino acid transporter and fatty acid binding proteins, and so forth, and were found in one day old larvae. Proteins of similar functions found in silk gland were 22 energy metabolism enzymes, 2 structural and processing proteins, and 2 transportation proteins. Seven enzymes like different subunits of cytochrome c oxidase, cytochrome c reductase, aldehyde dehydrogenase and ATP synthase subunits were found both in one day old larvae and silk gland transcriptome (Table 1 and Supplementary information).

#### 3.4.2. Genes Involve in Protein Synthesis

The proteins involved in protein synthesis and folding included 30 ribosomal proteins, 9 translational factors or tRNA/amino acid synthases, 10 chaperon-like proteins and 3 RNA polymerase or processing factors and were found in one day old larval library. In silk gland, 14 ribosomal proteins, 12 translational factors or tRNA/amino acid syntheses, 6 chaperons like proteins, 3 RNA synthesis and processing proteins were found. There was considerable difference in the expression of genes responsible for protein synthesis in these two libraries; only 7 ribosomal proteins and one hsp21.4 were found common in both libraries (Table 1 and supplementary information).

#### 3.4.3. Silk Proteins

**Figure 4:**
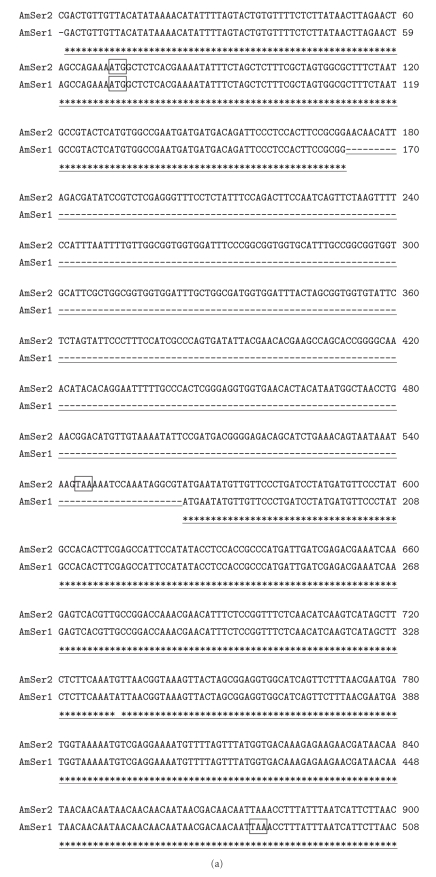

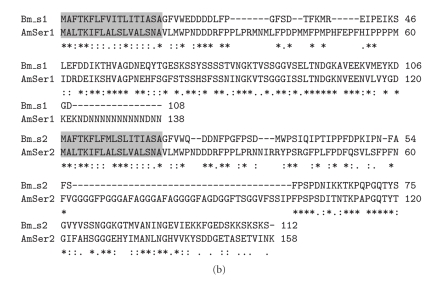
(a) Multiple sequence alignment of seroin genes (AmSer2 and AmSer1) of *A. mylitta*. The start and stop codons have been boxed as shown. Region underlined with “- - - -” indicates the exon absent in AmSer. (b) Protein sequence alignment of *A. mylitta* seroin1 (AmSer1) with *B. mori* Seroin1 (Bm_s1) [Genbank: AAL83945] and *A. mylitta* seroin 2 (AmSer2) with *B. mori* Seroin2 (Bm_s2) [Genbank: AAL83946]. The shaded area represents signal peptide sequence. The symbol “*” indicates identical residues, “:” indicates conserved substitution, and “.” indicates semi-conserved substitutions.

Fibroin, one of the major proteins of the silk of lepidopteran insects, has been studied extensively. This protein is produced in the posterior section of silk glands. Sericin, another protein of the silk is secreted in the middle section in addition to certain small molecular weight proteins. Our previous studies have indicated that *A. mylitta* fibroin is a homodimeric protein of two similar sized polypeptides of 197 kDa approximately and predominantly rich in amino acids like glycine, alanine and serine [23]. The complete fibroin gene of *A. mylitta* has not been cloned and sequenced. A cluster (Am[003]) comprised of 105 ESTs showed very high homology with the carboxy terminus of *Antheraea yamamai* fibroin (Table 1). Other six clusters showed homology with different regions of the fibroin gene from various silk moths but could not be assembled to any particular region of fibroin gene due to the repetitive nature of this gene sequence. No cluster showed homology with sericin, the second most abundant silk protein secreted in the silk gland has reported high level of sericin expression at the fifth instar stage [24]. The identification and characterization of the additional silk-specific small molecular mass protein, seroin, have been reported in *G. mellonella* [25] and *B. mori* [26]. A single seroin protein (147 amino acid residues) has been found in *G. mellonella* while two seroin proteins, seroin1 (108 amino acid residues) and seroin2 (112 amino-acid residues), encoded by two different genes, responsible for the protection of silk against moulds and bacteria, have been reported in *B. mori*. In this study we found two clusters, Am[001] (comprising 127 ESTs) and Am[021] (comprising 16 ESTs), whose deduced amino acid sequences showed homology to *B. mori *seroin1 with *E*-value 2 × *E* ^−06^ and 5.00 × *E* ^−07^ , respectively, and we designated them as AmSer2 and AmSer1, respectively. Both showed the presence of an identical N-terminal sequence (first 34 amino acid residues). The alignment of the nucleotide sequences of Am[001] coding for AmSer2 and Am[021] coding for AmSer1 showed exactly same sequences at the 5′ and 3′ ends with a region deleted in the middle in case AmSer2 (Figure 4(a)). These indicate production of seroin transcripts by alternative spilicing or gene duplication. Alignment of deduced amino acid sequences of *A. mylitta* and *B. mori* seroins although showed the high degree of similarity, several unique features were observed in AmSer1 and AmSer2. AmSer1 contained an asparagine rich C-terminus end (13 out of last 15 amino acid residues) absent in *B. mori* seroin1. AmSer2 has a glycine rich repetitive sequence not present in *B. mori* seroin2 (Figure 4(b)). A common feature between *B. mori* and *A. mylitta* seroins is the high degree of similarity in the signal peptide sequences. AmSer1 has a balanced ratio of non-polar and polar amino acids as seen in all seroins, but AmSer2 has twice as many non-polar residues as polar residues.

#### 3.4.4. Immune Related Genes

Several immune-related genes were identified in the present study. Various protease inhibitors, 8 Serpin-like proteins were found in the transcriptome of both the libraries (Table 1 and supplementary information). Serpins are implicated in prophenol oxidase activation, an innate immune response in anthropods [27]. Various proteases and protease inhibitors regulate the diverse mechanisms like melanization, phagocytosis and induction of anti-microbial peptides production [28]. They are produced in the middle section of the silk-secreting glands prior to cocoon spinning and their production is controlled at transcription level [26]. Such developmental expression pattern and the presence of proteinase inhibitors in the silk cocoon indicate that the functions of these proteins are linked to cocoon formation and maintenance. Hdd1 like protein and salivary secreted ribonuclease were found in one day old larvae. Beta glucosidase, O-glycosyl hydrolase, macrophage migration inhibitory factor, cyclophilin like proteins and serpins were present in the silk gland transcripts and may help in protection of cocoon from microbial infection (Table 1 and supplementary information).

### 3.5. Assignment of the *A. mylitta* Dataset to Gene Ontology Terms

**Figure 5:**
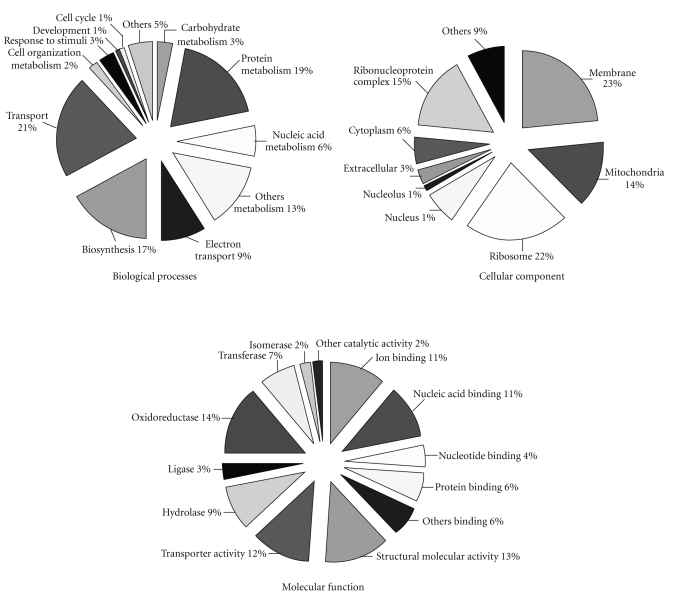
Representation of Gene ontology (GO) sub categorization of *A. mylitta* assembled ESTs.

Out of the 648 clusters, 353 showed a hit to the protein database Uniref90 as determined by BLASTX results using AutoFACT. In order to functionally categorize these clusters, Uniprot ID of each of these sequences was searched against QuickGO (http://www.ebi.ac.uk/ego/), the web based browser of the Gene Ontology data at the European Bioinformatics Institute. Two hundred and forty one out of the 648 clusters could be assigned to at least one GO accession number with a total of 1049 GO term assignments in the three main categories: 28% molecular function (290 out of 1049), 47% biological process (495 out of 1049), and 25% cellular component (264 out of 1049). Each cluster was further classified into one or more subcategories based on all the GO terms assigned to it. The total number of subcategories in molecular function, biological process and cellular component categories were twenty-nine, twenty-five and fifteen, respectively (Figure 5). The most abundant molecular function assigned was “binding” (38%) followed by “catalytic activity” (37%), “structural molecule activity” (13%) and “transporter activity” (12%). The most abundant subcategory in “binding” was “ion binding” and nucleic acid binding (11%) followed by “protein binding” (6%). The most abundant subcategory in “catalytic activity” was “oxidoreductase” (14%) followed by “hydrolase” (9%). The majority of the clusters were implicated in the biological process of “metabolism” (67%), followed by “transport” (21%), “response to stimulus” (3%), “cell organization and biogenesis” (2%), “cell cycle” (1%) and “development” (1%). The *A. mylitta* larva is polyphagous and consumes 100% of its food requirements during its larval stages to tide over the non-feeding pupa and adult stages [29]. Hence, the large number of metabolism related ESTs found here may be important in understanding this unique feature of silkmoths from the Saturdniidae family. Twenty one large subunit and eighteen small subunit ribosomal proteins were identified with high sequence accuracy because of the high EST redundancy associated with these genes. The most abundant cellular component subcategories assigned were “membrane” (23%), “ribosome” (22%), “ribonucleoprotein complex” (15%) and “mitochondrion” (14%) (Figure 5).

### 3.6. Comparative Genomics of *A. mylitta* Transcripts

**Figure 6:**
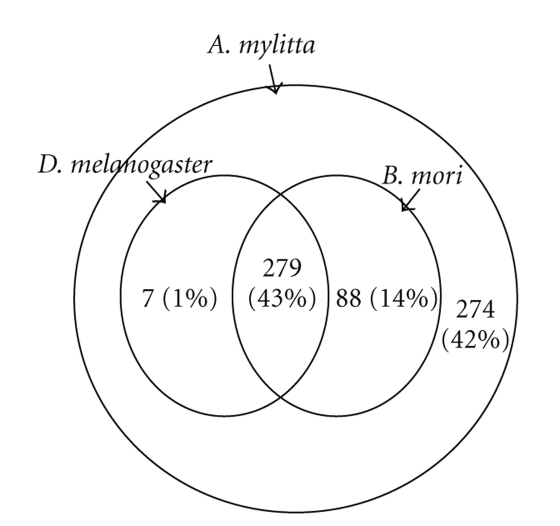
A Venn diagram showing the distribution of *A. mylitta* assembled sequences along with *D. melanogaster* and *B. mori* ESTs. The inner circles contain the numbers and percentages of *A. mylitta* ESTs that share similarity with *D. melanogaster* and *B. mori* ESTs. The region between inner circles and outer circle represents *A. mylitta* ESTs without any similarity with *D. melanogaster* and *B. mori* ESTs.

Comparative genomics help to trace gene evolution including the emergence, development, and loss of orthologous genes in different organisms over evolutionary time [30]. In this regard, we queried our assembled sequences to search for homologous sequences in the *D. melanogaster* and *B. mori* ESTs present in NCBI dbEST using tBLASTX algorithm (cut off *E*-value = 1*e* ^−06^). These species were chosen because they represent the most intensively studied dipteran and lepidopteran insects. The split between branches leading to Diptera and Lepidoptera were occurred 290–340 million years ago, a time of separation earlier than that between mammals and birds (International Lepidopteran Genome Project). However, 43% of the sequences had homology with both *D. melanogaster* and *B. mori* ESTs and probably represented non-dispensable genes normally conserved across the species. Fourteen percent of the sequences showed homology with only *B. mori* ESTs and probably identify lepidopteran specific genes (Figure 6). Apart from these, many sequences belong to the unclassified class, such as silk proteins, proteinase inhibitors, cuticle proteins, lipoprotein and hsp20.4 showed about 1% homology with only *D. melanogaster*. These may represent genes that were either lost from *B. mori* during evolution or those that may be present in the *B. mori* genome but not represented in the EST database. Finally, there were 274 (42%) sequences that had no tBLASTX match in the *D. melanogaster* and *B. mori* EST databases. Most of these include sequences that have been annotated in the class “unassigned protein” or “unclassified” (Figure 6).

These may be *Antheraea* specific genes forming an interesting set of ESTs for future research in *Antheraea* specific studies as well as comparison between mulberry (Bombycidae family) and non-mulberry silkworms (Saturdniidae family).

### 3.7. AmyBASE-EST Database for *A. mylitta*

A web accessible database called AmyBASE was created in order to store and navigate through the EST data. The database provides information about each cluster regarding its consensus sequence, length, EST sequences which make up the cluster, GO annotation, and homologous proteins/domains as identified by BLAST searches against various databases. Sequence details and library information for each EST are also available along with a cross reference to the cluster to which the EST belongs. An easy access to all these sequences associated with the various subcategories based on GO assignments in molecular function, biological process and cellular component categories has been provided. This will allow speedy retrieval of information about particular genes of interest. The option of BLAST-searching a nucleotide sequence against the clusters has also been made available to the user. The database can be freely accessed at http://www.btc.iitkgp.ernet.in/.

## 4. Conclusion

Here, we report the initial data set of transcript analysis undertaken for *A mylitta*. A total of 2476 ESTs obtained from this silkmoth resulted in 648 clusters and potentially represent 467 unigenes. A total of 437 clusters showed homology with known rRNA, protein, domain sequences or ESTs. Transcriptome analysis of one day old larvae and silk gland showed significant variation in expression of different genes and may lead to identify the silk gland specific genes. Further, expression and functional analysis of these novel genes may help to find certain genes unique to Lepidoptera species or *A. mylitta* in particular. Among the 648 clusters, 241 were categorized according to Gene Ontology (GO) hierarchy. Genes belonging to diverse subcategories have been found which will evoke multiple research interests in different areas of insect science. Of particular interest are the findings concerning *A. mylitta* fibroin and seroin, which call for further research into these commercially important genes. Finally, the entire EST data has been organized and made freely available at the online database, AmyBASE. It will provide users with an easy access to a comprehensive source of information via keyword search, BLAST query and GO functional annotations browsing capabilities. These EST data will provide an initial insight into the *A. mylitta* transcriptome and further aid in understanding the biological mechanisms and functions of lepidopteran species as well as insects in general.

## Figures and Tables

**Table 1:** A list of transcripts showing similarity with known proteins.

Cluster	No.	First Hit	Accession no.	*E*-value	Library information
(A) *Protein synthesis *					

(1) *Ribosomal proteins *					

Am[018]	18	60S acidic ribosomal protein P2 [*Spodoptera frugiperda*]	AAL62467	2*e* − 24	Silk gland
Am[022]	15	Ribosomal protein S28 [*Bombyx mori*]	NP_001037680	3.00*E* − 21	larvae (1d)

(2) *Translation factors, t-RNA/amino acid synthases *					

Am[033]	10	Glycyl-tRNA synthetase [*Bombyx mori*]	NP_001040293	5.00*E* − 152	Silk gland
Am[034]	10	Elongation factor 1 alpha [*Papilio xuthus*]	BAG30769	0.00*E* + 00	Silk gland

(3) *Chaperones, protein folding and secretion *					

Am[016]	20	Hsp70 [*Mythimna separata*]	ABY55233	3*e* − 142	larvae (1d)
Am[024]	15	Heat shock protein hsp21.4 [*Heliconius erato*]	ABS57447	1.00*E* − 16	Mixed

(4) *RNA synthesis and processing *					

Am[044]	8	Conserved hypothetical protein [*Nasonia vitripennis*]	XP_001604727	7.00*E* − 112	larvae (1d)
Am[157]	4	Transcriptional regulator, AraC family [*Pseudoalteromonas atlantica* T6c]	YP_660409	8	larvae (1d)
Am[351]	2	Activating transcription factor [*Bombyx mori*]	NP_001037041	8.00*E* − 34	Silk gland

(B) *House keeping *					

(1) *Energy metabolism *					

Am[005]	48	Cytochrome c oxidase subunit III [*Antheraea pernyi*]	NP_803442	2.00*E* − 94	Mixed
Am[009]	28	cytochrome c oxidase subunit I [*Antheraea yamamai*]	AAY78891	2*e* − 82	Mixed
Am[029]	11	NADH:ubiquinone dehydrogenase, putative [*Aedes aegypti*]	XP_001663929	1.00*E* − 56	larvae (1d)
Am[046]	8	ATP synthase F0 subunit 6 [*Antheraea pernyi*]	NP_803441	4.00*E* − 71	larvae (1d)
Am[078]	6	Phosphoribosyl pyrophosphate synthetase [*Bombyx mori*]	NP_001040481	2.00*E* − 104	larvae (1d)

(2) *Structural proteins *					

Am[012]	23	Troponin C 2 [*Lonomia obliqua*]	AAV91416	1*e* − 71	larvae (1d)
Am[057]	7	Flexible cuticle protein 12 precursor	P45589	4.00*E* − 34	larvae (1d)
Am[066]	6	Troponin I 1 [*Lonomia obliqua*]	AAV91417	1*e* − 132	larvae (1d)

(3) *Protein processing/degradation *					

Am[322]	2	Protease [*Helicoverpa armigera*]	ABU98623	5*e* − 42	larvae (1d)
Am[167]	3	Ubiquitin [*Antheraea yamamai*]	BAD05031	1.00*E* − 66	larvae (1d)

(4) *Transportation *					

Am[048]	8	Transport protein Sec61 gamma subunit [*Bombyx mori*]	NP_001037675	2.00*E* − 22	Silk gland
Am[137]	4	Vacuolar-type H+-translocating inorganic pyrophosphatase [*Plasmodium chabaudi chabaudi*]	XP_746228	0.2	larvae (1d)
A0149F	1	Fatty acid-binding protein 2 (FABP 2)	P31417	1*e* − 30	larvae (1d)

(C) *Other categories *					

(1) *Detoxification *					

Am[023]	15	Slucosyl/glucuronosyl transferases [*Tribolium castaneum*]	XP_973188	7.00*E* − 60	Silk gland
Am[039]	9	Carboxylesterase [*Bombyx mori*]	NP_001040411	6.00*E* − 37	larvae (1d)
Am[049]	8	Glutathione S-transferase theta [*Antheraea pernyi*]	ACB36909	2*e* − 100	Silk gland

(2) *Regulation *					

Am[052]	8	Activated C kinase 1 receptor [*Choristoneura fumiferana*]	AAZ29605	0	Silk gland
Am[098]	5	Cyclic nucleotide-specific phosphodiesterase long isoform [*Plasmodium falciparum* 3D7]	ABS50256	3.40*E* + 00	larvae (1d)

(3) *Immune related proteins/protease inhibitors *					

Am[007]	43	Protease inhibitor 3 [*Lonomia obliqua*]	AAV91425	6.00*E* − 74	Silk gland
Am[019]	17	Hdd1-like protein [*Trichoplusia ni*]	ABV68857	5*e* − 31	larvae (1d)
Am[037]	9	Pacifastin light chain precursor [Pacifastacus leniusculus]	AAC64661	2.00*E* − 13	larvae (1d)
Am[062]	7	Serpin-like protein [*Antheraea mylitta*]	ABG72716	2*e* − 48	Silk gland

(4) *Silk proteins *					

Am[001]	127	Seroin 2 [*Antheraea mylitta*]	ABG72728	8.00*E* − 48	Silk gland
Am[002]	119	Fibroin heavy chain [*Antheraea yamamai*]	AAL02118	1*e* − 15	Silk gland
Am[021]	16	Seroin 1 [*Bombyx mori*]	NP_001037045	4.00*E* − 07	Silk gland

(5) *Other functions *					

Am[004]	74	Polyprotein [Kakugo virus]	YP_015696	3.00*E* − 150	Silk gland
Am[027]	12	Chemosensory protein 9 [*Bombyx mori*]	NP_001037066	3.00*E* − 25	larvae (1d)

*1d indicates one day old larvae.

## Abbreviations

EST: Expressed Sequence Tag
NCBI: National Centre for Biotechnology Information
dbEST: Database Expressed Sequence Tags (NCBI).
